# Prevalence and factors associated with depressive symptoms among adolescents in Saudi Arabia after the COVID-19 pandemic: a cross-sectional study

**DOI:** 10.3389/fpsyt.2026.1878623

**Published:** 2026-07-01

**Authors:** Moustafa A. Hegazi, Duaa H. Abudawoud, Turki S. Alahmadi, Waleed A. Alghamdi, Ali F. Atwah, Manal M. Almazrui, Raghad A. Alahmadi, Muhannad M. Alshuwayki, Adi A. Alzahrani, Abdulrahman S. Alqahtani, Alabbas Horaib, Batool A. Al Hussain

**Affiliations:** 1 Department of Pediatrics, Faculty of Medicine, King Abdulaziz University, Rabigh, Saudi Arabia; 2 Department of Pediatrics, Mansoura University Children’s Hospital, Mansoura, Egypt; 3 Department of Pediatrics, Faculty of Medicine, King Abdulaziz University, Jeddah, Saudi Arabia; 4 Department of Psychiatry, Faculty of Medicine, King Abdulaziz University, Jeddah, Saudi Arabia; 5 Faculty of Medicine, King Abdulaziz University, Rabigh, Saudi Arabia; 6 College of Medicine, Vision Colleges, Riyadh, Saudi Arabia

**Keywords:** after the COVID-19 pandemic, among adolescents, depressive symptoms, factors, prevalence, Saudi Arabia

## Abstract

**Background and objectives:**

Adolescent depression has risen globally with substantial increase during COVID-19 pandemic compared with pre-pandemic levels due to multiple reasons including quarantine and school closures. This study aimed to determine the prevalence and factors associated with depressive symptoms among adolescents in Saudi Arabia after the acute phase of the COVID-19 pandemic and its associated disruptions.

**Methods:**

A multiregional cross-sectional online survey enrolled adolescents (10–18 years) in Saudi Arabia. Depressive symptoms were assessed using Patient Health Questionnaire–Adolescent version (PHQ−A). Data from PHQ-A, demographic and psychosocial data were analyzed by univariate and multivariate regression comparing adolescents with and without depressive symptoms to identify significant factors.

**Results:**

A total of 518 adolescents with a mean age of 16.60 years participated; 355 (68.5%) had depressive symptoms (mild 27.4%, moderate 19.5%, moderately severe 13.7%, severe 7.9%) and 163 adolescents (31.5%) reported no or minimal symptoms. In multivariate regression, the significant independent factors associated with depressive symptoms, were dissatisfaction with facial appearance (AOR = 3.80, p<0.001), major life challenges (AOR = 3.39, p<0.001), excellent academic level (AOR = 3.12, p=0.001), low self−esteem (AOR = 2.41, p=0.006), being in secondary/high school (AOR = 2.24, p=0.006), and academic stress (AOR = 2.10, p=0.006). Other factors identified in univariate analysis lost significance in the multivariate model. The regression model demonstrated an overall accuracy/predictability of 80.9% with excellent discrimination between adolescents with and without depressive symptoms.

**Conclusions:**

This is one of few comprehensive assessments of recent prevalence and wide-range of factors associated with depressive symptoms among adolescents. Depressive symptoms were high among adolescents even after the COVID-19 pandemic, consistent with global patterns. Psychological/personal factors including dissatisfaction with appearance, major life challenges and academic pressure were more significantly associated with depressive symptoms than sociodemographic factors. These findings can guide targeted awareness campaigns and preventive measures in Saudi Arabia and similar global settings. There is a need for identifying adolescents with depressive symptoms to provide culturally appropriate interventions including school-based screening programs, counseling services, family/teachers’ engagement and accessible mental health support with particular attention to self−image concerns and academic stress.

## Introduction

Depression is a prevalent and serious mental health disorder that adversely influences emotional state, thinking pattern, and behavior. It is a mood disorder characterized by persistent sadness, reduced interest or pleasure in usual activities, and disturbances in regulatory functions such as appetite and sleep (1).

Adolescence is a critical developmental stage characterized by biological, psychological, and social transitions that can increase vulnerability to mental disorders, including depression. Academic demands, identity formation, and shifting social roles may generate sustained stress. Prior research has linked adolescent depression with academic pressure, high school/college entrance exams, low parental educational attainment, limited family income, family conflicts, and early maternal childbearing age (2). Recent data also suggest an upward trend in adolescent depression in several settings, making it an increasingly important global concern for schools and health systems (2, 3).

Before COVID-19 pandemic, clinically significant depressive symptoms in large youth cohorts were estimated at approximately 12.9%. The COVID-19 pandemic created prolonged school closures, reduced peer interaction, quarantine measures, and increased family stress, all of which can precipitate psychological distress in youth. Evidence also suggests that pandemic-related deterioration in parental mental health may indirectly affect children and adolescents and increase risk of maltreatment in stressed households (4–8). A meta-analysis reported pooled estimates of depressive symptoms of 25.2% and doubled prevalence of depression during the pandemic than pre-pandemic levels, particularly among older adolescents and girls (4). In addition, lockdown-related lifestyle changes, including sleep disruption, dietary habits changes, decreased physical activity, and increased digital media use, were associated with worse mental health among adolescents (9).

In Saudi Arabia, reported prevalence rates of adolescent depression vary between 14.3% and 38.2%, with identified risk factors including female gender, socioeconomic adversity, family and peer relationship difficulties, exposure to violence, and personal or family psychiatric history (10–12). A national study conducted across all 13 regions in 2015 reported a prevalence of 14.3% among 12, 575 adolescents (13). In Riyadh prior to COVID-19, moderate to severe depressive symptoms were reported in 32.4% of intermediate and secondary school students, with female gender, lower paternal education, and exposure to abuse emerging as significant predictors (14).

Data on adolescent depression in Saudi Arabia during and after COVID-19 remain limited. Evidence from the pandemic period reported high depressive symptom prevalence among university students (15), while post-pandemic research highlighted clinically relevant depressive symptoms among adolescents with behavioral vulnerabilities such as internet gaming disorder (16). Given the evolving post-pandemic context, periodic national-level assessments of depressive symptoms and associated risk factors remain essential.

It is expected that the prevalence of depression in adolescents may have been decreased after global regression of COVID-19 pandemic. In a study from China, after regression of COVID-19 pandemic, depression rates ranged from 9.29%-13.05%, among middle and high school students. Significant associations with gender, smoking, alcohol use, and spending more than four hours online daily, were detected (2).

Periodic national-level assessments of depressive symptoms and associated risk factors in the highly susceptible adolescents remain essential. Therefore, this study aimed to assess the recent prevalence and main factors associated with depressive symptoms among adolescents in Saudi Arabia after the acute phase of the COVID-19 pandemic and its associated disruptions that may guide early detection and targeted prevention.

## Methods

### Selection of participants

This cross−sectional analytical study assessed depressive symptoms among adolescents aged 10–18 years living in Saudi Arabia after the acute phase of COVID−19 pandemic. Data were gathered by an online self−administered survey distributed across the five regions of Saudi Arabia using convenience snowball sampling. Recruitment of participants started at 01/11/2024 and ended at 31/03/2025. The survey link and invitation message were distributed through social media platforms (WhatsApp, X, Instagram, and Facebook) and through institutional and community networks. Because the survey was disseminated through open online channels, the number of individuals exposed to the invitation could not be determined and a response rate was not calculated. Of the participants who accessed the survey, 82.4% completed all mandatory questions. To prevent duplicate participation, the used questionnaires were converted to Google forms to restrict responses to registered accounts (e.g., one response per each Google account) and all responses were screened for potential duplicates before analysis. Adolescents outside the specified age range, living outside Saudi Arabia, or providing incomplete responses were excluded.

Ethical approval was provided by the Research Ethics Committee, Faculty of Medicine in Rabigh, King Abdulaziz University (Reference No: 24028). All methods were performed in accordance with the relevant guidelines and the declaration of Helsinki. Participation was voluntary and anonymous.

Both adolescent assent and written informed consent from their legal guardians were obtained prior to participation in this online survey. Given the anonymous nature of data collection, participants could not be individually identified, directly contacted, or referred for intervention. Before participation, respondents were informed that some questions addressed sensitive topics, including depressive symptoms and suicidal thoughts. Participation was entirely voluntary, and respondents could skip any question or withdraw from the survey at any time.

Participants were informed that, because of the survey’s anonymity, the research team would be unable to provide immediate direct assistance or intervention if suicidal ideation or intent was disclosed. Information on crisis-support resources and suicide-risk response procedures was provided at both the beginning and end of the survey. In accordance with the recommendations accompanying the PHQ-A, participants and their legal guardians were advised to seek immediate professional assistance from local emergency services or mental health providers, including psychiatric hospitals, general hospitals, outpatient psychiatric clinics, and primary health-care centers, if the participant reported severe depressive symptoms or serious suicidal thoughts or suicide attempts.

### Measuring instrument/tool (questionnaire)

Depressive symptoms were assessed using the Patient Health Questionnaire–Adolescent version (PHQ-A), which was made available in both Arabic and English to allow accessibility for a broader range of participants, including non-Saudi adolescents who may not speak Arabic. The Arabic version of the PHQ has been validated in a Saudi population and was found to be a reliable and valid screening tool for depression (17).

The PHQ-A is similar to the PHQ-9 but includes 4 additional questions. The additional PHQ-A items were translated from English into Arabic through a forward-translation process conducted by two bilingual experts who were native Arabic speakers and fluent in English. The Arabic version was then back-translated into English by two independent bilingual translators who were blinded to the original questionnaire. The resulting Arabic PHQ-A was pretested in a pilot study to assess its clarity, comprehensibility, and cultural appropriateness and to identify any necessary modifications. Data obtained from the pilot study were not included in the final analysis.

PHQ−A includes nine items scored 0–3 (total score 0–27) and additional 4 unscored questions, with higher scores indicating greater symptom severity (18). PHQ-A is a reliable tool which demonstrated good screening performance in adolescents (19, 20). The survey also collected sociodemographic data and a broad range of established factors associated with depressive symptoms in adolescents including academic, lifestyle, and psychosocial/behavioral factors.

### Sample size determination

Sample size estimation was performed before study initiation to ensure adequate statistical power. The minimum required sample size was estimated to be 385 adolescents based on an assumed prevalence of depressive symptoms of 50%, a 95% confidence level, and a 5% margin of error. Adolescents aged 10–18 years constitute approximately 14% of the Saudi Arabian population (about 5 million individuals). The final sample exceeded this requirement, supporting the adequacy of the study to estimate the prevalence of depressive symptoms with acceptable precision. Sample size estimation was performed using the Raosoft sample size calculator (https://raosoftcalculator.com/).

### Data scrutinization and statistical analysis

Statistical analyses were conducted by IBM SPSS version 25. Data were checked for completeness and inconsistencies and each completed questionnaire was double checked for clarity of responses by 2 members of the research team before data entry. Categorical variables were summarized as frequencies and percentages and compared using χ2 or Fisher’s exact tests; continuous variables were summarized as mean ± SD and compared using independent−samples t−tests. Univariate logistic regression was used to estimate unadjusted odds ratios (OR) with 95% confidence intervals. Univariate logistic regression analysis was performed to estimate unadjusted odds ratios (ORs) and their corresponding 95% confidence intervals (CIs). Candidate predictors for the multivariable logistic regression model were selected based on evidence from previous scientific literature and systematic reviews, focusing on clinically relevant and theoretically established factors associated with adolescent depression. The selected variables included sociodemographic, academic, lifestyle, and psychosocial/behavioral variables, as well as variables that were statistically significant in the univariate analysis. Multivariable logistic regression was then conducted to estimate adjusted odds ratios (AORs) and identify the independent factors associated with depressive symptoms. Statistical significance was set at p<0.05.

## Results

A total of 518 adolescents provided complete responses. According to PHQ-A scores, 355 participants (68.5%) were categorized as having depressive symptoms ranging from mild to severe, while 163 (31.5%) had no or minimal symptoms.

**Table 1** shows the prevalence of depressive symptoms across severity levels. Mild, moderate, moderately severe, and severe depressive symptoms were identified in 27.4%, 19.5%, 13.7%, and 7.9% of the participating adolescents, respectively.

**Table 1:** Prevalence of depressive symptoms in participating adolescents.

PHQ-A score	Frequency (n)	%
0–4 No or minimal	163	31.5
5–9 Mild	142	27.4
10–14 Moderate	101	19.5
15–19 Moderately severe	71	13.7
20–27 Severe	41	7.9
Total	518	100.0

Several demographic and socioeconomic factors were significantly linked with depressive symptoms among adolescents in univariate analysis (**Table 2**). Female gender (p=0.007), non-Saudi nationality (p=0.005), monthly income of family less than 10, 000 SAR (p=0.038), financial troubles (p= 0.001), rented house (p=0.04), and living with one parent or alone (p=0.03) showed higher prevalence of depressive symptoms. Father’s lower education level (p=0.013) also emerged as a significant risk factor.

**Table 2:** Univariate analysis comparing demographic, socioeconomic, housing, family, and academic characteristics between adolescents with and without depressive symptoms.

Variables	All participants (n=518)		Adolescents with depressive symptoms (n=355)		Adolescents without depressive symptoms (n=163)		OR (95% CI)	p value
	N	%	N	%	N	%		
Demographic characteristics								
Gender								
Male* Female	219 299	42.3 57.7	136 219	38.3 61.7	83 80	50.9 49.1	1.67 (1.15-2.43)	0.007
Region								
Central East West North South	95 122 110 93 98	18.3 23.6 21.2 18.0 18.9	73 66 87 64 65	20.6 18.6 24.5 18.0 18.3	22 56 23 29 33	13.5 34.4 14.1 17.8 20.2	NA	0.0003
Nationality								
Saudi* Non-Saudi	478 40	92.3 7.7	319 36	89.9 10.1	159 4	97.5 2.5	4.49 (1.57-12.82)	0.005
Socioeconomic characteristics								
Monthly income of family (SAR)								
More than 10, 000* Less than 10, 000	332 186	64.1 35.9	217 138	61.1 38.9	115 48	70.6 29.4	1.52 (1.02-2.27)	0.038
Financial troubles/problems								
No* Yes	400 118	77.2 22.8	260 95	73.2 26.8	140 23	85.9 14.1	2.22 (1.35-3.67)	0.001
Mother’s education level								
University and Postgraduate studies* Secondary (high) school or less	308 210	59.5 40.5	203 152	57.2 42.8	105 58	64.4 35.6	1.36 (0.92-1.99)	0.12
Father’s education level								
University and Postgraduate studies* Secondary (high) school or less	302 216	58.3 41.7	194 161	54.6 45.4	108 55	66.3 33.7	1.63 (1.11-2.40)	0.013
Mother’s occupation								
Employed* Unemployed (house wife)	213 305	41.1 58.9	139 216	39.2 60.8	74 89	45.4 54.6	1.29 (0.89-1.88)	0.18
Father’s occupation								
Employed* Unemployed	513 5	99.0 1.0	351 4	98.9 1.1	162 1	99.4 0.6	1.85 (0.20-16.65)	0.58
Housing characteristics								
House								
Own* Rented	366 152	70.7 29.3	241 114	67.9 32.1	125 38	76.7 23.3	1.56 (1.02- 2.38)	0.04
Type of house								
Front view house or Villa with garden* Popular house or back view house	366 152	70.7 29.3	247 108	69.6 30.4	119 44	73.0 27.0	1.18 (0.78- 1.79)	0.43
Family characteristics/living condition								
Both parents* One parent, with a relative or alone	441 77	85.1 14.9	294 61	82.8 17.2	147 16	90.2 9.8	1.91 (1.06-3.42)	0.03
Academic characteristics								
Academic grade								
Intermediate school* Secondary (high) school	152 366	29.3 70.7	86 269	24.2 75.8	66 97	40.5 59.5	2.13 (1.43-3.16)	0.0002
Academic level/performance								
Weak or Average* Excellent	123 395	23.7 76.3	88 267	24.8 75.2	35 128	21.5 78.5	0.84 (0.54-1.32)	0.45
Academic stress	334	64.5	261	73.5	73	44.8	3.42 (2.32-5.05)	0.000
Dissatisfaction with academic achievement	220	42.5	180	50.7	40	24.5	3.16 (2.09-4.78)	0.000

*Reference category.

**Table 3** presented multiple characteristic personal and psychological features, contributing as significant factors linked with depressive symptoms among adolescents in univariate analysis. Depressive symptoms were significantly associated with academic stress (p<0.001), irregular physical activity (p<0.001), poor friendship quality (p<0.001), exposure to bullying (p<0.001), excessive use of mobile/internet (p<0.001), dissatisfaction with facial appearance (p<0.001), body image dissatisfaction (p<0.001), poor social skills (p<0.001), dissatisfaction with academic achievement (p<0.001), low self-esteem (p<0.001), family member previously diagnosed with depression (p=0.002), poor relation and communication with mother/father (p<0.001), staying inadequate time with parents (p<0.001), participation in theft or property destruction (p=0.01), smoking (p=0.02) and exposure to major life challenges (p<0.001).

**Table 3:** Univariate analysis comparing lifestyle, peer, personal, psychological, family and behavioral characteristics between adolescents with and without depressive symptoms.

Variables	All participants (n=518)		Adolescents with depressive symptoms (n=355)		Adolescents without depressive symptoms (n=163)		OR (95% CI)	p value
	N	%	N	%	N	%		
Lifestyle and Screen-Time Factors								
Irregular sport/exercise (< 3 d/W)	308	59.5	230	64.8	78	47.9	2.01 (1.38-2.92)	0.0003
Mobile and internet use > 4 h/d	350	67.6	258	72.7	92	56.4	2.05 (1.3-3.03)	0.0003
TV watching > 4 h/day	42	25.1	27	7.6	15	9.2	4.49 (1.57-12.82)	0.54
Video games play > 3 h/day	130	25.1	93	26.2	37	22.7	1.21 (0.78-1.87)	0.39
Peer Relationship and Social Factors								
Lack of close friend (s)	44	8.5	35	9.9	9	5.5	1.87 (0.88-3.99)	0.10
Poor friendship quality	203	39.2	162	45.6	41	25.2	2.50 (1.66-3.77)	0.000
Exposure to verbal/physical bullying	86	16.6	73	20.6	13	8.0	2.99 (1.60-5.57)	0.0006
Self-Perception and Psychological Factors								
Unsatisfied or hate body image	251	48.5	197	55.5	54	33.1	2.52 (1.71-3.71)	0.000
Dissatisfaction with facial appearance	201	38.8	184	51.8	17	10.4	9.24 (5.37-15.92)	0.000
Dissatisfaction with social skills	268	51.7	222	62.5	46	28.2	4.25 (2.84-6.35)	0.000
Dissatisfaction with personal opinions, decision-making abilities (Low self-esteem)	229	44.2	196	55.2	33	20.2	4.86 (3.14-7.51)	0.000
Personal &Family Medical/Mental Illness								
Previously diagnosed with depression	36	6.9	28	7.9	8	4.9	1.66 (0.74-3.72)	0.22
Previously diagnosed with other chronic illness (organic-mental-psychiatric)	11	2.1	9	2.5	2	1.2	2.09 (0.45-9.80)	0.34
Family member previously diagnosed with depression	60	11.6	52	14.6	8	4.9	3.33 (1.54-7.18)	0.002
Family member previously diagnosed with other illness (organic-mental-psychiatric)	24	4.6	21	5.9	3	1.8	3.35 (0.99-11.41)	0.053
Family Relationship Factors								
Staying inadequate time with parents<8h/d	183	35.3	146	41.1	37	22.7	2.38 (1.56-3.63)	0.0001
Poor relation/communication with father	180	34.7	163	45.9	17	10.4	7.29 (4.23-12.56)	0.000
Poor relation/communication with mother	112	21.6	103	29.0	9	5.5	6.99 (3.44-14.23)	0.000
Major life challenges/Stressful Life Events								
Experienced extreme challenges, difficulties, or major life-changes	280	54.1	237	66.8	43	26.4	5.61 (3.71-8.47)	0.000
Exposure to physical abuse	31	6.0	26	7.3	5	3.1	2.50 (0.94-6.63)	0.058
Exposure to sexual abuse	10	1.9	7	2.0	3	1.8	1.07 (0.27-4.20)	0.92
Behavioral and Substance Use Factors								
Participation in theft/property destruction	31	6.0	28	7.9	3	1.8	4.57 (1.37-15.25)	0.01
Smoking	57	11.0	47	13.2	10	6.1	2.33 (1.15-4.75)	0.02
Drugs/narcotics abuse	6	1.2	4	1.1	2	1.2	0.92 (0.17-5.06)	0.92
Alcohol consumption	10	1.9	6	1.7	4	2.5	0.68 (0.19-2.46)	0.56

The multivariable logistic regression model included 25 candidate predictors and 355 outcome events, yielding an events-per-variable (EPV) ratio of 14.2. This exceeded the traditional minimum recommendation of 10 events per variable and approached more conservative recommendations of 15 EPV, showing adequate power of the multivariate model.

Multi-collinearity among independent variables was assessed using variance inflation factors (VIFs) and tolerance statistics before running the multivariable regression analysis. No evidence of problematic multicollinearity was observed, with all VIF values below 5 and tolerance values above 0.20.

The multivariate regression analysis (**Table 4**) identified the following as the most significant independent factors associated with depressive symptoms in descending order; dissatisfaction with facial appearance (AOR = 3.80, p<0.001), major life challenges (AOR = 3.39, p<0.001), excellent academic level (AOR = 3.12, p=0.001), low self-esteem (AOR = 2.41, p=0.006), being in secondary/high school (AOR = 2.24, p=0.006) and academic stress (AOR = 2.10, p=0.006).

**Table 4:** Multivariate logistic regression for factors associated with depressive symptoms in adolescents.

Variables	Significance			95% C.I.	
	B	P value	AOR	LCL	UCL
Demographic factors					
Female gender (Male*)	0.51	0.08	1.66	0.95	2.90
Non-Saudi nationality (Saudi*)	1.09	0.08	2.96	0.87	10.11
Socioeconomic factors					
Low family income <10, 000 SAR/mon (>10, 000 SAR/mon*)	0.24	0.44	1.27	0.70	2.31
Financial troubles (No troubles*)	0.18	0.60	1.20	0.62	2.32
Rented house (Own house*)	0.20	0.50	1.22	0.68	2.19
Father education of high school level or less (University and Postgraduate studies*)	0.31	0.26	1.36	0.80	2.34
Academic factors					
Secondary or high school (Intermediate school*)	0.81	0.006	2.24	1.26	3.99
Academic stress (No stress*)	0.74	0.006	2.10	1.24	3.54
Excellent academic level (Weak or average academic level*)	1.14	0.001	3.12	1.55	6.28
Dissatisfaction with academic achievement (Satisfied*)	0.22	0.51	1.25	0.65	2.42
Family factors					
Living alone or with one parent (Living with both parents*)	-0.07	0.86	0.93	0.41	2.09
Family member diagnosed with depression (No history of depression*)	0.29	0.55	1.33	0.52	3.41
Staying inadequate duration with parents (Adequate stay*)	0.29	0.30	1.34	0.77	2.34
Poor relation and communication with father (Good relation*)	0.72	0.05	2.06	0.99	4.30
Poor relation and communication with mother (Good relation*)	0.15	0.75	1.16	0.46	2.90
Relation with peers and social factors					
Poor friendship quality (Good quality*)	0.28	0.33	1.33	0.75	2.33
Exposure to bullying (No exposure to bullying*)	0.34	0.39	1.41	0.65	3.08
Poor social skills (Good social skills*)	0.21	0.49	1.23	0.69	2.19
Lifestyle and behavioral factors					
Irregular sport (less than 3 d/w) (Regular sport*)	0.27	0.30	1.31	0.79	2.18
Use of mobile/internet > 4 h/d (Use of Use of mobile/internet< 4 h/d*)	0.43	0.11	1.53	0.91	2.57
Smoking (No smoking*)	0.46	0.34	1.59	0.61	4.11
Self-Perception and Psychological Factors					
Dissatisfaction with body image (Satisfied*)	-0.31	0.27	0.74	0.43	1.27
Dissatisfaction with facial appearance (Satisfied*)	1.34	<.001	3.80	1.88	7.69
Low self-esteem (High self-esteem*)	0.88	0.006	2.41	1.29	4.52
Major life challenges or stressful life events (No major challenges*)	1.22	<.001	3.39	1.98	5.78

AOR, adjusted odds ratio; CI, confidence interval; LCL, lower confidence limit; UCL, upper confidence limit. *Reference categories.

**Figure 1:**
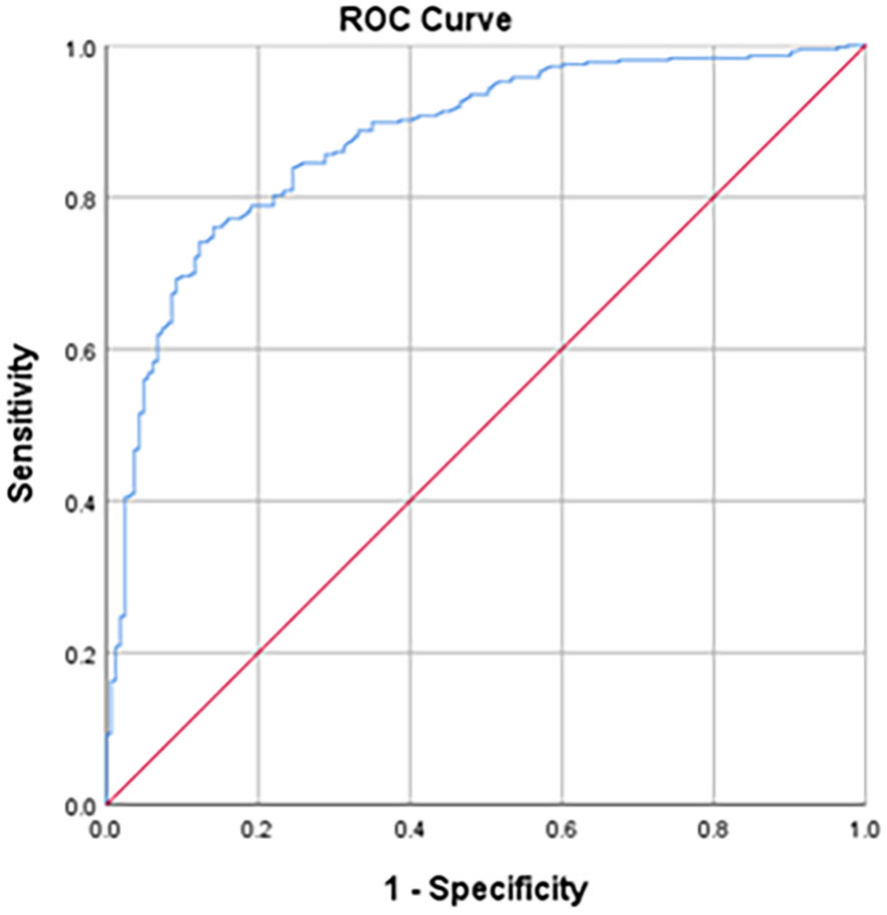
Receiver operating characteristic curve for the multivariable logistic regression model predicting screen-positive depressive symptoms. AUC = 0.875, 95% CI [0.843, 0.907].

The logistic regression model demonstrated good predictive performance with an overall accuracy/predictability of 80.9%. Sensitivity was 86.5%, indicating strong ability to correctly identify adolescents with depressive symptoms, while specificity was lower at 68.7%. The receiver operating characteristic (ROC) analysis further showed that the entire regression model allowed reliable and strong distinction between adolescents with and without depressive symptoms with excellent discrimination area under curve (AUC) of 0.875, with a precise estimate (SE = 0.016), strong statistical significance (p<0.001), and a narrow confidence interval (95% CI: 0.843–0.907). Hosmer–Lemeshow test was used to evaluate the goodness-of-fit of the regression model (χ2 = 9.86, degree of freedom = 8, p = 0.275), indicating that the model demonstrated acceptable fit. ROC is presented in **Figure 1**.

The response to additional unscored questions of PHQ-A revealed that 61.6% of adolescents felt depressed or sad most days in the past year, 13.7% of adolescents had serious suicidal thoughts in the past month, and 12.5% of adolescents had attempted suicide at least once in their lifetime.

## Discussion

This multi-regional survey detected a high prevalence of depressive symptoms in 68.5% of adolescents, including 21.6% with moderately severe or severe symptoms. Although the overall proportion appears higher than some earlier local estimates, differences in measurement tools, sampling methods, and study periods limit direct comparison. For example, a Riyadh study in 2019 among adolescent females reported a prevalence of approximately 30% (12). However, another Riyadh-based study using the PHQ-9 identified moderate to severe depression in 32.4% of adolescents which is even significantly more than 13.7% rate of moderately severe depression in our adolescents (14). High burden of depressive symptoms during the COVID-19 period closely similar to our findings has also been reported among science college students in Riyadh (15).

Internationally, comparable prevalence of depressive symptoms of 67.5% has been described during pandemic-related restrictions in Bangladesh (21), whereas a study from Morocco reported higher rates of moderately severe to severe depression symptoms of 44.7% (22). A comparative meta-analysis supported a sustained increase in depression after the pandemic compared with pre-pandemic estimates (5). Thus, other studies have reported an even higher prevalence of moderate to severe depressive symptoms among adolescents, underscoring the alarmingly high persistent global burden (including Saudi Arabia) of depression before, during, and after the COVID-19 pandemic. These findings support our results, indicating that adolescents worldwide, continue to experience significant rates of depression, particularly in the post-COVID-19 era.

In univariate analysis, depressive symptoms were associated with several sociodemographic factors including female gender, non-Saudi nationality, father’s lower educational level, maternal unemployment, economic status, living space/condition consistent with prior national and international studies (12, 14, 15, 23–26).

The findings in univariate analysis (**Table 3**) linked poor friendships, poor family relationships and communication with mother/father, bullying, low self-esteem and low social skills with depressive symptoms consistent with the findings in other Saudi (12, 14, 27) and international studies (28, 29). However, more physical activity and less internet use were considered preventive against depression (14). Additionally risky behaviors such as smoking were associated with increased depression, consistent with findings from studies in China and South Africa (2, 20). However, multivariable modelling identified psychological and contextual factors as the strongest independent factors of depressive symptoms among adolescents, rather than traditional sociodemographic variables. Key factors included dissatisfaction with facial appearance, major life challenges, academic stress among high school students, and low self-esteem. Appearance-related dissatisfaction and negative body image have previously been linked to depressive symptoms among Saudi adolescents (12, 30). Major life challenges as COVID-19 pandemic may reflect cumulative stressors, including pandemic-related disruptions and their consequences, which have been associated with increased depressive symptoms globally (4, 29).

A notable and interesting finding was the independent association between excellent academic performance and depressive symptoms. While low academic performance (<60%) has been identified as a main risk factor in a Chinese study (24), accumulating evidence suggests that academic pressure, perfectionism, and worry from failure can contribute to emotional distress and depression among high-achieving adolescents (31, 32). Studies of gifted or high-ability adolescents indicated that higher cognitive ability does not necessarily protect against depression and may be accompanied by social isolation or heightened expectations (33, 34). One multination study including 3955 adolescents from Hong Kong, Taiwan, the UK, and the Netherlands, showed that high-ability, female, and Western adolescents had higher levels of depression than their counterparts (35). School-based approaches that emphasize mastery-oriented goals rather than competitive performance may therefore reduce depression risk (36). Thus, high cognitive abilities and striving for perfection do not always shield gifted adolescents who may set unrealistically high standards for themselves from the pressures of academic achievement and they can sometimes heighten the risk for mental health issues, including depression. This may be due to social isolation, high expectations, social comparison and exacerbation of the feelings of inadequacy or loneliness resulting from disconnection from peers.

Low self-esteem was also an independent predictor, aligning with evidence that self-esteem plays a central role in adolescent resilience and later mental health outcomes (35, 37, 38). Difficulties in identity development have been associated with depressive symptoms, and bidirectional relationships between identity processes and depression have been reported in adolescents and emerging adults (39–43). These findings suggest that dissatisfaction with personal opinions and decision-making abilities may represent an important psychological pathway contributing to adolescent depression.

Another concerning result of this study was the proportion of adolescents reporting persistent sadness (61.6%) in the past year which is close to 68.5% of adolescents found to have depressive symptoms in this study. Also, lifetime suicide attempts are clinically concerning, particularly within the Saudi context where stigma surrounding mental health may limit disclosure and help-seeking. Similar trends in youth mental health outcomes have been reported internationally during the COVID-19 era (44). These findings highlight immediate necessity for culturally sensitive, school-based mental health screening, early counselling pathways, and family engagement, with specific attention to academic stress and self-image concerns.

This study is limited by its cross-sectional design hindering cause-effect determination, dependence on self-reporting, online convenience sampling that may limit representativeness and possible underrepresentation of adolescents outside the school system. Additional limitations include non-response bias, digital-access bias, social desirability bias, limited validation of certain psychosocial items, absence of sampling weights, limited generalizability to all Saudi adolescents and lack of current-sample PHQ-A reliability assessment. Moreover, PHQ-A is considered only as a screening tool to detect depressive symptoms in adolescents without confirmed diagnosis, so, the findings of this study should be cautiously interpreted with need for further in-depth assessment and follow up of identified adolescents with depressive symptoms.

Nevertheless, this study has many strengths including a multi-regional sample, adequate study power, assessment of a broad range of sociodemographic and psychosocial factors, and multivariable modelling that enables prioritization of independent factors for helpful targeted prevention. This study is also strengthened by being one of few comprehensive assessments of a broad range of factors associated with depressive symptoms among Saudi and non-Saudi adolescents, use of a validated simple, brief self-administered standardized screening tool (PHQ-A) allowing comparison with other national and global findings. Additionally, in-depth systematic statistical analyses and robust multivariate analyses with adjustment for confounders allowed the estimation of AOR, determination of effect size/role of each factor associated with depressive symptoms in a descending order of frequency. The regression model demonstrated strong predictive performance and excellent discrimination between adolescents with and without depressive symptoms.

## Conclusions

This study is among few comprehensive assessments of recent prevalence and wide-range of factors associated with depressive symptoms among adolescents in Saudi Arabia. The burden of psychological problems like depression on adolescents was great during COVID-19 pandemic due to many reasons including quarantine or lockdown, school closure, modification of learning and examination systems and disconnection from peers/social life. However, even after the end of the COVID-19 pandemic, there is a rising prevalence of depressive symptoms among adolescents with majority of adolescents experiencing varying degrees of depressive symptoms in Saudi Arabia which is consistent with global patterns. The observed prevalence of screen-positive depressive symptoms was high in this multi-regional online sample. However, direct inference about temporal change is limited by differences in sampling, measurement, and study period across available studies. Psychological and personal factors, including academic pressure especially in high school students, dissatisfaction with appearance, and exposure to major life challenges, were more significantly associated with depressive symptoms than sociodemographic factors in this survey.

## Recommendations

The findings of this survey can guide targeted awareness campaigns and preventive measures in Saudi Arabia and similar global settings. There is a need for identifying adolescents with depressive symptoms to provide culturally appropriate interventions including school-based screening programs, counseling services, family/teachers’ engagement and accessible mental health support with particular attention to self−image concerns and academic stress. Families and teachers need training to recognize early warning signs and provide support. Future longitudinal research is recommended to clarify causal pathways and guide targeted preventive strategies.

Large population-based cohort studies are required to investigate whether excellent academic level/academic stress especially in high school adolescents is a causal risk factor that should be targeted in school- and policy-based interventions. Schools, parents, and mental health professionals need to be mindful of the emotional burden/strain associated with academic pressure, even on the most academically successful students, emphasizing the need for balanced academic environment that promote both achievement and mental well-being.

Additionally, large-scale national and international studies are required to address the relation between depressive symptoms and dissatisfaction with facial appearance or experiencing major life challenges.

## Data Availability

The original contributions presented in the study are included in the article/supplementary material. Further inquiries can be directed to the corresponding author.
